# Scaling up for end-to-end on-chip photonic neural network inference

**DOI:** 10.1038/s41377-025-02029-z

**Published:** 2025-09-17

**Authors:** Bo Wu, Chaoran Huang, Jialong Zhang, Hailong Zhou, Yilun Wang, Jianji Dong, Xinliang Zhang

**Affiliations:** 1 Wuhan National Laboratory for Optoelectronics, School of Optical and Electronic Information, Huazhong University of Science and Technology, Wuhan, 430074 China; 2 Department of Electronic Engineering, The Chinese University of Hong Kong, Shatin, Hong Kong SAR China

**Keywords:** Integrated optics, Silicon photonics

## Abstract

Optical neural networks are emerging as a competitive alternative to their electronic counterparts, offering distinct advantages in bandwidth and energy efficiency. Despite these benefits, scaling up on-chip optical neural networks for end-to-end inference is facing significant challenges. First, network depth is constrained by the weak cascadability of optical nonlinear activation functions. Second, the input size is constrained by the scale of the optical matrix. Herein, we propose a scaling up strategy called partially coherent deep optical neural networks (PDONNs). By leveraging an on-chip nonlinear activation function based on opto-electro-opto conversion, PDONN enables network depth expansion with positive net gain. Additionally, convolutional layers achieve rapid dimensionality reduction, thereby allowing for an increase in the accommodated input size. The use of a partially coherent optical source significantly reduces reliance on narrow-linewidth laser diodes and coherent detection. Owing to their broader spectral characteristics and simpler implementation, such sources are more accessible and compatible with scalable integration. Benefiting from these innovations, we designed and fabricated a monolithically integrated optical neural network with the largest input size and the deepest network depth, comprising an input layer with a size of 64, two convolutional layers, and two fully connected layers. We successfully demonstrate end-to-end two-class classification of fashion images and four-class classification of handwritten digits with accuracies of 96% and 94%, respectively, using an in-situ training method. Notably, performance is well maintained with partially coherent illumination. This proposed architecture represents a critical step toward realizing energy-efficient, scalable, and widely accessible optical computing.

## Abstract (web summary)

This work presents a scalable on-chip optical neural network leveraging partially coherent sources for real-valued computing and wavelength-multiplexed parallelism, enhancing accessibility and integration potential.

## Graphical abstract

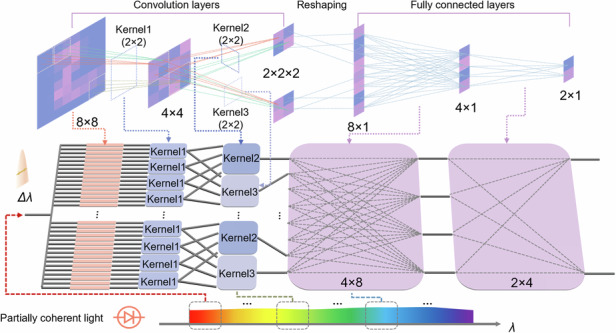

## Introduction

Artificial intelligence is a driving force behind technological advancements, with neural networks serving as its foundational cornerstone. However, these networks require substantial energy to operate, underscoring their significant power consumption^1^. Optical neural networks (ONNs), proposed within the last decade, have emerged as a promising alternative to their electronic counterparts^2^. This promise lies in their inherent advantages, including the low energy consumption of optical propagation and access to high-bandwidth resources across multiple parallel dimensions^3,4^. End-to-end ONNs refer to architectures that directly map input information to inference results in the optical domain^5,6^. This streamlined approach eliminates the need for separate preprocessing, information relay, or post-processing stages in the electrical domain, thereby reducing energy consumption and latency. Various paradigms of ONNs have been proposed, broadly categorized into spatial diffraction-based and on-chip schemes^7^. Spatial diffraction-based networks enable connections among millions of spatial sampling points in the weight matrix^8–10^. However, they are typically bulky, with a low integration density, and their performance is constrained by the refresh rate of spatial light modulators or digital micromirror devices, which is usually limited to kilohertz or megahertz-level frequencies^8^. In contrast, on-chip schemes leverage linear weight implementations through mechanisms such as Mach-Zehnder interferometer (MZI) meshes^11–15^, micro-ring resonator (MRR) weight banks^16,17^, crossbar arrays^18,19^, or on-chip diffraction^12,20–22^. These on-chip implementations overcome the refresh rate limitations of spatial diffraction schemes, achieving significantly higher rates—up to tens of gigahertz^23^.

**Fig. 1:**
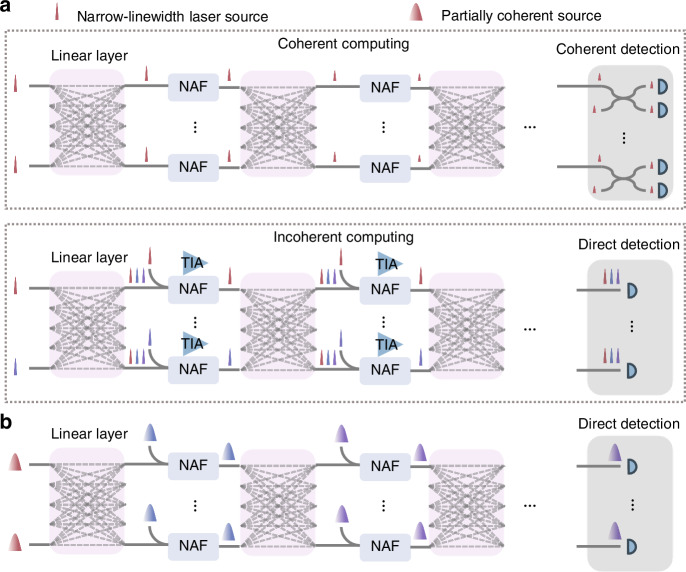
Comparison of scaling limitations in existing ONNs and the proposed solution. **a** Scaling challenges in current on-chip ONNs, including depth and input size limitations. The depth limitation arises from NAFs lacking net gain or requiring electrical amplification. The input size limitation is constrained by the need for coherent detection in coherent computing and multiple wavelengths in incoherent computing. **b** The proposed partially coherent deep optical neural network, which overcomes these limitations by enabling scalable depth and input size expansion

Although significant efforts have been made to develop various architectures for on-chip ONNs, the majority of these designs utilize the optical chip merely as an operator within a complex neural network model, falling short of achieving end-to-end optical inference. Recent advancements have made significant strides in end-to-end ONNs, showcasing exceptional energy efficiency and minimal latency^5,6^. However, scaling up these architectures remains challenging due to constraints on network depth and the limited size of the on-chip optical matrix (Fig. 1a). Nonlinear activation functions (NAFs) are essential for the flexible functionality of deep neural networks and can be implemented in either the electrical or optical domain. Electrical nonlinearity involves converting signals between optical (analog) and electrical (digital) domains, which not only precludes end-to-end optical inference but also introduces significant energy consumption and latency^24^. A competent optical NAF must meet several key requirements, including cascadability (offering positive net gain), compatibility with massive production processes, low energy consumption, and high bandwidth^25–30^. Among various integrated schemes, opto-electro-opto (O-E-O) nonlinearity stands out due to its strong cascadability and compatibility with scalable production processes^5,6,17^. However, demonstrations of on-chip deep ONNs to date have either failed to achieve positive net gain or have relied on external electrical amplifiers, limiting their efficiency and scalability^5,6^. Besides, the size of the on-chip optical matrix is constrained by the inherent integration density of optical devices and the reliance on narrow-linewidth lasers. Narrow-linewidth lasers require either an increased number of wavelengths for incoherent computing or scalable coherent detection for coherent computing, producing additional complexity^6,31,32^. In contrast, partially coherent optical sources offer significant advantages, such as reduced feedback control and thermal management requirements, greater parallelism, and enhanced robustness^32^. These benefits facilitate the scaling up of on-chip optical matrices.

In this work, we introduce a partially-coherent deep optical neural network (PDONN) chip to overcome the scaling up issue (Fig. 1b). The architecture comprises an input layer with a record scale of 64, two convolutional layers, and two fully connected layers. The convolution layers enable rapid data dimensionality reduction, effectively overcoming the limitations of input size. The proposed O-E-O nonlinearity facilitates scalable cascading across multiple hidden layers with positive net gain. Notably, we demonstrate that PDONN can effectively utilize a partially coherent source for both linear and nonlinear operations, achieving performance comparable to the same architecture employing a narrow-linewidth laser diode. Furthermore, the network is configured with an in-situ training method, which enhances its robustness against fabrication errors and improves its generalization ability across different applications. Our results demonstrate excellent end-to-end classification accuracy on benchmark tasks, underscoring the advantages of PDONN in terms of computational power, energy efficiency, and adaptability. This advancement highlights the potential of the proposed architectures for next-generation optical computing applications.

## Results

### Principle of the PDONN chip

**Fig. 2:**
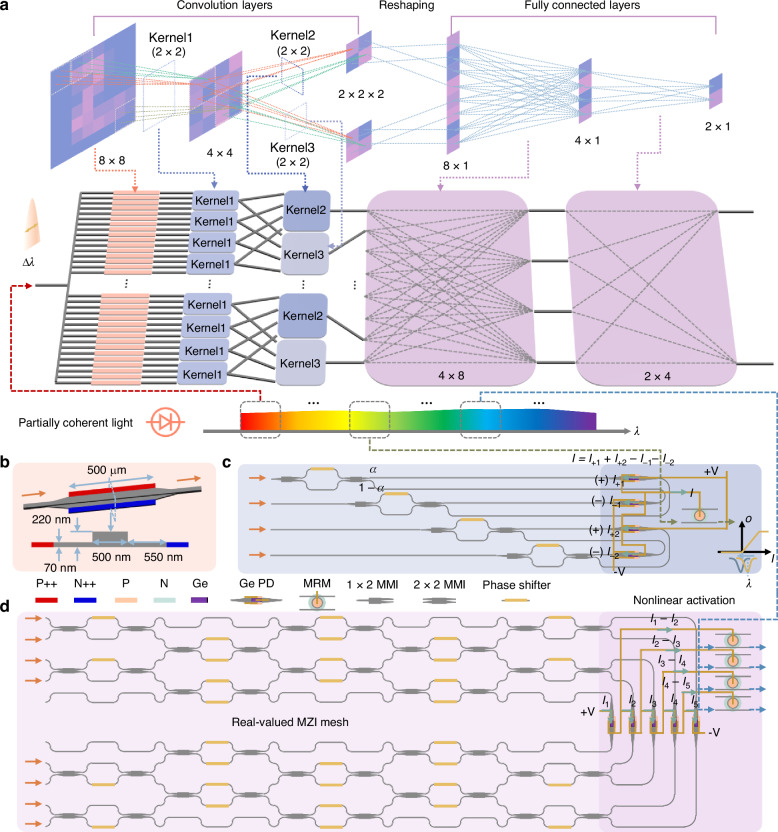
The overall architecture of the proposed optical neural network. **a** The network comprises one input layer, two convolutional layers, and two fully connected layers. The optical source is generated by an LED or amplified spontaneous emission (ASE) source, characterized by a wavelength linewidth of Δ*λ*. **b** The intensity modulator (IM) based on carrier injection encodes the input information onto the optical source. The IM features a typical P-I-N doping profile and has a total length of 500 μm. **c** The optical dot-product unit, acting as the convolution kernel, splits the optical signal into positive and negative components, which are directed to separate photodetectors. The resulting differential photocurrent drives the micro-ring modulator (MRM) to produce the required nonlinearity. **d** The first fully connected layer consists of a 4 × 8 Mach–Zehnder interferometer (MZI) matrix and the corresponding optical nonlinear activation function (NAF). The 4 × 8 MZI matrix is decomposed into two 4 × 4 MZI matrices, and their outputs are summed through multi-port photodetectors. The differential photocurrent drives the MRM to generate nonlinearity

The mathematical framework of the proposed optical deep neural network is illustrated in Fig. 2a. The process begins with an input image (8 × 8), which is convolved with a kernel (2 × 2) using a stride of 2. After the NAF is applied, the image’s dimensions are reduced to 4 × 4. Subsequently, two kernels of the same size (2 × 2) are separately applied to the 4 × 4 image, producing an 8 × 1 vector that serves as the input to two fully connected layers. The output vector, where the highest value indicates the classification result, completes the end-to-end inference. Overall, this architecture is a standard convolutional neural network, and the following sections detail its implementation on a fully-integrated silicon photonic chip.

The input image is reshaped into a one-dimensional vector (64 × 1) and encoded onto the optical source. To enable high-speed refreshing of input information, an intensity modulator (IM) based on carrier injection is employed as the information encoder (Fig. 2b). The core component of the convolutional layer is a real-valued optical dot-product unit, depicted in Fig. 2c. The input optical signal is split into positive and negative channels based on the kernel’s value, using a thermally tunable MZI. Specifically, the normalized kernel value is defined as *κ* = 2*α*-1where *α* represents the ratio of optical power entering the positive channel. By adjusting the thermal phase shifter in one arm of the MZI, *κ* can be tuned to any value within the range [−1, 1]. The light in the positive and negative channels is directed to corresponding photodetectors connected in a differential configuration. According to the Kirchhoff’s current law, the differential photocurrent is calculated as *I* = *I*_+1_ + *I*_+2_ − *I*_–1_ − *I*_–2_ and it represents the convolution result. This photocurrent directly drives a micro-ring modulator (MRM) to achieve nonlinearity. When the injected current *I* > 0, the MRM is forward-biased, causing a significant blue shift in the transmission spectrum as *I* increases. Conversely, for *I* < 0, the MRM is reverse-biased, resulting in a slight redshift due to the lower carrier-depletion modulation efficiency^33^. The output light intensity from the MRM’s through port follows a linearized sigmoid function relative to the driving current. The intensity of the supply light determines the gain of the NAF, and a positive net gain is achieved when the intensity reaches a sufficiently high level, indicating that the PNONN can be effectively cascaded.

The fully connected layer is implemented using a simplified real-valued MZI mesh, as shown in Fig. 2d. The 4 × 8 MZI matrix is decomposed into two 4 × 4 MZI matrices, with the resulting optical intensities summed via multi-port photodetectors^34^. This customized MZI mesh is optimized for incoherent optical inputs, minimizing the number of phase shifters and eliminating redundancy^35^. It outputs the differential optical power as a real-valued result, with cascaded differential photodetectors processing the signals. Similar to the convolutional layer, the nonlinear optical response is achieved by feeding the differential current to an MRM.

The optical source used for input and for powering the NAF is partially coherent, with a linewidth Δ*λ*, and can be realized using a light-emitting diode (LED) or amplified spontaneous emission (ASE) source (see Supplementary Information S1). Unlike narrow-linewidth laser sources, it only requires direct detection and does not limit the number of input channels in the optical matrix, thereby facilitating the scaling up of ONNs^32^. To fully exploit the ultra-broadband wavelength resources, different wavelength bands are directed into different optical layers. The proposed PDONN operates in the real-valued domain, enabling the direct representation of both positive and negative weights without requiring additional encoding. This reduces hardware complexity and energy consumption, and facilitates scaling up the network size. Real-valued computation is particularly advantageous under partially coherent illumination, where representing signed values through phase manipulation becomes challenging^32^.

### Chip fabrication and characterization

**Fig. 3:**
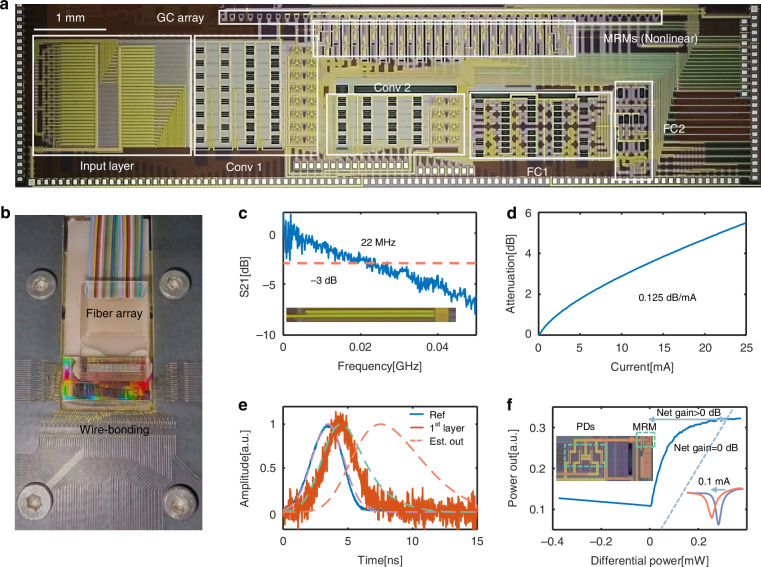
Fabrication and characterization of the PDONN chip. **a** Microscope image of the PDONN chip, showing the grating coupler (GC) array used for optical input and output coupling. Conv1/2 represents the 1st/2nd convolution layer, and FC1/2 represents the 1st/2nd fully connected layer. **b** The packaged PDONN chip with integrated optical and electrical interfaces. **c** Measured S21 parameter of the on-chip IM, showing a 3-dB bandwidth of 22 MHz. **d** Attenuation coefficient of the IM as a function of the injected current. **e** Delay measurements for the first nonlinear layer of the PDONN chip, along with the estimation of the final output signal. Dashed lines indicate fitted waveforms for better visualization. **f** Typical NAF utilized in the experiment, demonstrating its effectiveness. Dotted line denotes the transfer function with 0 dB net gain

The PDONN chip was fabricated in a standard silicon photonics foundry, with its microscope image presented in Fig. 3a. The total chip footprint is approximately 17 mm^2^, integrating hundreds of optical devices within this compact area. Figure 3b highlights the packaged PDONN chip, equipped with optical and electrical interfaces to facilitate experimental testing. The on-chip IM was first calibrated, as shown in Fig. 3c, d. The measured 3-dB bandwidth of the IM is 22 MHz, significantly exceeding the bandwidth of traditional thermally tuned MZI encoders. The modulation efficiency was determined to be 0.125 dB/mA. For encoding purposes, a current range of 0 mA to 23 mA was selected, corresponding to an attenuation range of 0 dB to 5.18 dB. The latency of the chip was characterized by comparing the optical pulse through a reference waveguide (230 μm in length) with the output pulse of the first nonlinear layer, as shown in Fig. 3e. The latter pulse exhibited an additional latency of approximately 1 ns, attributed to signal propagation within silicon waveguides, metal wiring, and the RC delay of the O-E-O nonlinearity. Signal propagation accounted for about 100 ps, while the RC latency, modeled as a first-order system, was approximately 1.67 ns (dashed line, see Supplementary Information S2 for details). Under the same input pulse, the total latency of the PDONN chip after three nonlinear layers was estimated as 4.1 ns, comprising 3.7 ns from NAFs and 400 ps from signal propagation. Figure 3f illustrates the optical NAF response as a function of varying differential input powers, with the center wavelength resonating at zero input power. The saturable optical power was measured as only 0.2 mW, reflecting high energy efficiency. In addition, the reference 0 dB-net-gain line (dotted line) is shown on the plot, with the vertical axis in mW. The measured transfer function, above the zero-net-gain line, demonstrates a positive net gain, confirming that the PDONN can be efficiently cascaded.

### Image classification results

**Fig. 4:**
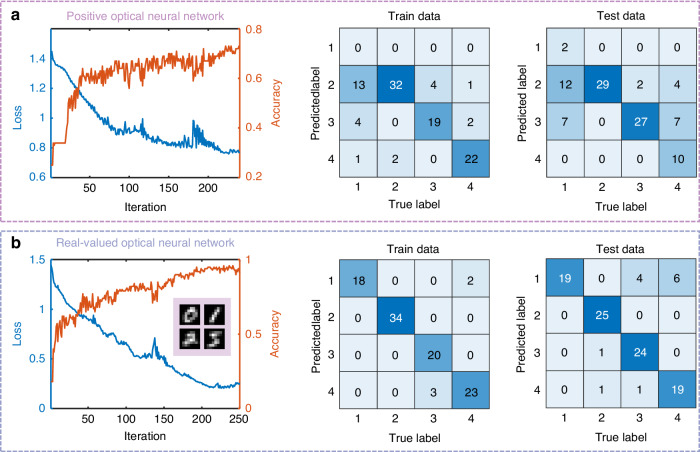
The results on the 4-classification of handwritten digits. **a** Positive optical neural network. **b** Real-valued optical neural network

Using the same chip, we can flexibly deploy either 4-classification or 2-classification task based on the selected output channels. In our experiment, the drop ports of the MRMs at the first fully connected layer serve as the output channels for the 4-classification task. For the 2-classification task, the power differences across three ports in the final layer represent the computing results (see Supplementary Information S3). The softmax function is applied to process the outputs from the PDONN, and the cross-entropy loss function is used for training the network. A gradient descent algorithm is employed for in-situ training of the PDONN (see “Methods”). It treats the PDONN as a black box, making it inherently robust to fabrication errors and environmental disturbances, thereby enhancing its generalization ability across various applications. We first use the performance of the ONN with a narrow-linewidth optical source as a reference. The wavelengths of the laser sources are slightly detuned to achieve equivalent incoherent linear computing with the MZI mesh^25^. For the 4-classification task, we use the MNIST dataset as the benchmark, selecting 100 images containing the handwritten digits “0”, “1”, “2”, and “5” as the training set. More datasets can enhance the generalization capability of the model and reduce overfitting. The images are resized to 8 × 8 pixels using bilinear interpolation before being loaded onto the on-chip IMs. Another set of 100 images from the dataset is used for testing. To implement a positive ONN, we disconnect the negative photodetectors in the convolution layers from the electrical bias system, while maintaining the optical NAF of the fully connected layer in the real domain. The training process and the final confusion matrix of the positive ONN are shown in Fig. 4a. The classification accuracy for the training and testing datasets is 73% and 68%, respectively. For the same 4-classification task, the accuracy of the real-valued ONN is 95% and 87% for the training and test datasets, respectively (Fig. 4b). These results demonstrate that the real-valued ONN outperforms the positive ONN.

**Fig. 5:**
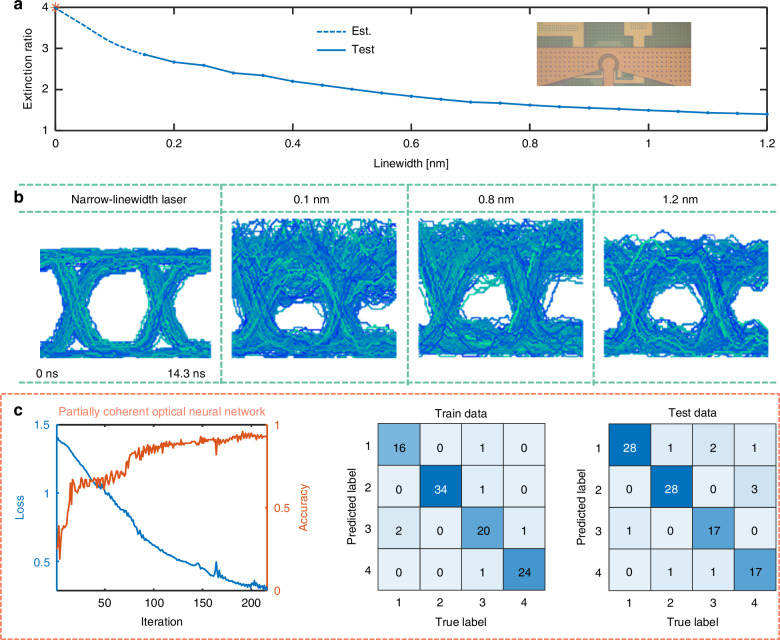
Demonstration of partially coherent optical neural network. **a** Extinction ratio of the MRM as a function of the linewidth of the input partially coherent optical source. The star marks the extinction ratio with a narrow-linewidth laser input, while the dashed line represents the estimated value. **b** Measured eye diagram of the MRM for partially coherent optical sources with different linewidths at an operating frequency of 140 MHz. **c** The results on the 4-classification of handwritten digits with the partially coherent optical neural network

After benchmarking with narrow-linewidth lasers, we further validate the PDONN’s robustness by employing a partially coherent optical source. The performance of the optical NAF using a partially coherent source depends on balancing the extinction ratio of the MRM with the noise level of the source (see Supplementary Information S1). The extinction ratio of the MRM was studied as a function of the linewidth of the partially coherent source (Fig. 5a). As the linewidth increased, the extinction ratio decreased sharply, adversely affecting the expressiveness of the ONN. To evaluate modulation quality, the eye diagrams of the MRM were measured at a modulation rate of 140 MHz with varying linewidths of partially coherent sources (Fig. 5b). With increased linewidth, the eye diagram quality initially improved due to increased noise frequency but would deteriorate with further increases, caused by the lower extinction ratio. Consequently, a linewidth of 0.4 nm was selected to balance noise and extinction ratio, enabling optimal performance for the PDONN. In the experiment, a wavelength-selective switch partitions the ASE source into four wavelength bands with linewidth of 0.4 nm, each of which enters one layer of ONNs. The optical paths of the different channels of supplying light are carefully configured to ensure that the path difference exceeds the coherence length of the optical source. The intensity of the input light is maintained at the same level as in the previous experiment. For the four-classification task, the partially coherent ONN achieves high accuracies of 94% and 90% for the training and test datasets, respectively (Fig. 5c). These results suggest that the moderate degradation of the extinction ratio in the nonlinear layer, caused by the partially coherent source, does not significantly impair the overall performance of the ONN. Results for the 2-classification task can be found in the Supplementary Information S3.

## Discussion

### The multiplexing technology of the PDONN

**Fig. 6:**
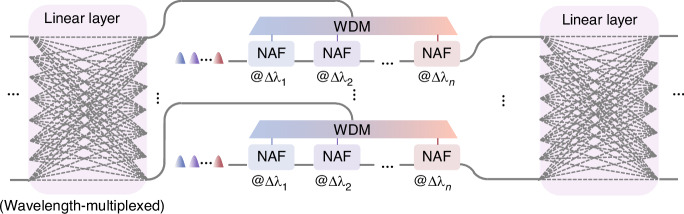
The multiplexing technology of the proposed PDONN

Compared to coherent optical sources, partially coherent optical sources offer significantly higher parallelism in wavelength dimensions^32^. This advantage paves the way for a promising architecture of a wavelength-multiplexed deep ONN, as illustrated in Fig. 6. Leveraging the broadband feature of the linear computing layer enables the processing of multiple wavelength channels in parallel^36^. With carefully designed optical paths, the MZI mesh can maintain cascadability without compromising its broadband characteristics (see Supplementary Information S1). In the nonlinear layer, information is first demultiplexed into individual wavelength channels and converted into photocurrent. Subsequently, this photocurrent drives MRMs resonating at distinct wavelength bands to achieve the corresponding NAF. The multiplexing functionality of MRMs aggregates multi-channel information into a bus waveguide, which delivers the nonlinear results to the next linear layer. To avoid inter-channel crosstalk, the wavelength bands must be spectrally separated. For denser wavelength utilization, the bus waveguide can be replaced by a wavelength division multiplexer (WDM) to efficiently combine the different wavelength channels with less crosstalk.

### Key performance comparison of different ONNs

The classification performance of our fabricated PDONN is primarily limited by the extinction ratio of data loading and optical NAF, both of which present promising avenues for improvement. The low extinction ratio of the fabricated MRM (6 dB) constrains the signal-to-noise ratio of the partially coherent source when using a linewidth of only 0.4 nm in the experiment. There are two viable optimization strategies for accommodating moderate extinction ratios with larger linewidths. The first approach is to enhance the intrinsic extinction ratio of the MRM, which can readily exceed 10 dB with optimized fabrication techniques^37^. Another promising direction is to increase the free spectral range (FSR) of the on-chip modulator. The current fabricated MRM has a radius of approximately 10 μm, corresponding to an FSR of nearly 12 nm. By adopting nanocavity-based optical modulators, it is feasible to achieve an FSR-free spectrum with a broad 3-dB optical bandwidth, providing sufficient spectral range to support partially coherent optical sources with linewidths exceeding 1 nm^38^. In our experiments, only the simple MNIST dataset and a limited set of 100 training samples were used. We anticipate that more complex datasets and larger training sets can be explored with further scaling of the network and improvements in data communication speed.

Latency and energy efficiency are two critical metrics of an ONN. The pulse latency per inference for the fabricated chip is estimated as 4.1 ns. The system’s bandwidth is primarily determined by the RC bandwidth of the optical NAF, calculated as approximately 105 MHz, corresponding to an RC latency of 1.67 ns. With optimized parasitic parameters in the optical modulator and photodetector, the bandwidth could extend to the GHz range, reducing latency to as low as 100 ps^39^. The data-loading speed, currently limited by the IMs, could be significantly enhanced by adopting carrier-depleted modulators^23^. The power consumption of the system is derived from three main sources: the laser source, external electrical circuits, and the optical chip. In total, the power consumption of the experimental system is 10.57 W (see Supplementary Information S4 for details). For computational operations, a dot product involves 2*N* operations, where *N* is the vector size, and each NAF is equivalent to three additional operations. For the PDONN, the total operations per inference are 356 OPs. The energy efficiency is calculated as (based on the current chip without optimization): 10.57 W × 4.1 ns/356 Ops = 121.7 pJ/OP. Increasing the size of the optical matrix could further enhance energy efficiency^40^.

Table 1 compares the PDONN with state-of-the-art on-chip ONNs. This work demonstrates the largest input size and number of optical layers, while maintaining low latency and high energy efficiency. Optical NAFs with a net gain greater than 0 dB facilitate the scalable cascading of multiple optical layers on a single chip. The latency of 4.1 ns is higher than that of other on-chip schemes, due to the use of wider optical pulses and the inclusion of RC latency in the NAFs. However, the 400 ps propagation latency remains comparable to previous works that rely on O-E-O nonlinearity. Moreover, this work represents the first demonstration of partially coherent real-valued computing using an on-chip ONN, alleviating the reliance on narrow-linewidth lasers. This relaxation in source requirements enhances the scalability of the on-chip optical matrix while maintaining high inference performance.

**Table 1:** Comparison of different on-chip ONNs

Architecture	Input size	Num. of optical layers	Latency	Energy efficiency	Optical source	Operation domain
Ref. ^42^	15	1	N.A.	144.2 pJ/OP	Coherent	Positive
Ref. ^6^	6	3	410 ps	9 pJ/OP	Coherent	Complex
Ref. ^5^	30	3	570 ps	14 pJ/OP	Coherent	Positive
This work	64	4	4.1 ns^a^	121.7 pJ/OP^a^	Partially coherent	Real

^a^RC latency of NAF is considered in every inference

In conclusion, we propose a scaling up strategy for on-chip ONNs by employing cascadable optical NAFs, an efficient convolution architecture, and a partially coherent optical source. These innovations collectively enable exceptional scalability and significantly enhance the performance of widely accessible ONNs. We successfully designed and fabricated a cutting-edge deep ONN chip, comprising two convolutional layers and two fully connected layers. Experimental evaluations demonstrated strong performance in both 4-class and 2-class classification tasks, underscoring the advantages of real-valued computing and the partial coherence of the optical source. Future improvements, such as enhancing the extinction ratio of modulators and further reducing system latency, will enable even better scalability and performance. With its groundbreaking design and demonstrated capabilities, the proposed PDONN is a strong candidate for large-scale artificial intelligence applications, marking a significant step toward the development of energy-efficient and scalable optical computing systems.

## Materials and methods

### Dataset

The dataset of MNIST handwritten digits and MNIST fashion products was respectively taken from https://yann.lecun.com/exdb/mnist/ and https://github.com/zalandoresearch/fashion-mnist.

### In-situ training algorithm

We use the gradient descent algorithm to train the ONN, aiming to minimize the mean entropy loss of the training dataset by optimizing the voltages applied to the thermal phase shifters. The detailed training process is as follows:

**1. Initialization**: Randomly initialize the voltages applied to the thermal phase shifters.

**2. Gradient approximation**:

- Increment the voltage by 0.05 V for each thermal phase shifter and calculate the corresponding loss function *L*(*U* + 0.05).
- Decrement the voltage by 0.05 V for each thermal phase shifter and calculate the loss function *L*(*U* − 0.05).
- Estimate the approximate gradient of the loss function using the formula:

1 $$G=\frac{L(U+0.05)-L(U-0.05)}{0.1}$$

**3. Voltage update**: Update the voltages using the Adam algorithm, a fast-converging gradient descent method, as described by the formula^41^:

2 $$\begin{array}{c}U({\rm{iter}}+1)=U({\rm{iter}})-\alpha ({v}_{{\rm{iter}}}/(1-{{\beta }_{1}}^{{\rm{iter}}}))/\sqrt{{s}_{{\rm{iter}}}/(1-{{\beta }_{2}}^{{\rm{iter}}})+\varepsilon }\\ {v}_{{\rm{iter}}}={\beta }_{1}{v}_{{\rm{iter}}-1}+(1-{\beta }_{1})G\\ {s}_{{\rm{iter}}}={\beta }_{2}{s}_{{\rm{iter}}-1}+(1-{\beta }_{2}){G}^{2}\end{array}$$

where iter is the current iteration number, *α* is the learning rate which is set to 0.05 during training, *β*_1_, *β*_2,_ and *ε* are hyperparameters set to 0.9, 0.999, and 10^–8^, respectively. The initial values of *v*_iter_ and *s*_iter_ are zero.

**4. Iteration**: Repeat steps 2 and 3 until the loss function converges.

**5. Save optimized parameters**: Once the training is complete, save the optimized voltages for deployment.

This gradient-based training process effectively fine-tunes the ONN for high-performance computation.

### Experiment methods

The chip was fabricated using a 200 mm CMOS process line with a two-layer copper interconnect and a small line width of 130 nm. The on-chip photodetectors employ a lateral PIN structure with a 260 nm-thick germanium layer. Calibration of the MRMs on the chip was achieved by sweeping the thermal phase shifters integrated into them. The bandwidth of the on-chip IMs was measured using a 20 GHz vector network analyzer (Anritsu MS2028C). The eye diagram of the MRMs was recorded using a setup comprising an arbitrary waveform generator (GIGOL DG4202, 500 MSa/s), a photodetector with 200 MHz bandwidth, and an oscilloscope (GIGOL DS4022, 4 GSa/s). For chip latency measurements, the input laser source was modulated using a lithium niobate intensity modulator with 10 GHz bandwidth. The pulse signal was generated with an arbitrary waveform generator (GIGOL DG4202), and the output optical signal was detected by an 18 GHz bandwidth photodetector. The detected signal was captured by an oscilloscope (Tektronix DSA72004B). The thermal phase shifters and the bias voltage of the optical NAFs were driven by a digital-to-analog converter (LTC2688) controlled by a field-programmable gate array (FPGA) chip (7K325T). The entire experimental setup was controlled via a personal computer through serial ports, and the chip was thermally stabilized using a thermoelectric cooler (TEC).

## Supplementary information

Supplementary information for Scaling up for end-to-end on-chip photonic neural network inference

## Data Availability

The datasets used and/or analyzed in the current study are available from the corresponding author upon reasonable request.
