# Supplementary material for: Scaling up for end-to-end on-chip photonic neural network inference
Source: Light Sci Appl. 2025 Sep 17;14:328. doi: 10.1038/s41377-025-02029-z (PMC12443992; doi:10.1038/s41377-025-02029-z)
Supplement: Supplementary file 1 — Supplementary information for Scaling up for end-to-end on-chip photonic neural network inference [file 41377_2025_2029_MOESM1_ESM.docx]

**Supplementary information for “Scaling up for end-to-end on-chip photonic neural network inference”**

Bo Wu^1^, Chaoran Huang^2^, Jialong Zhang^1^, Hailong Zhou^1, *^, Yilun Wang^1^, Jianji Dong^1, *^ and Xinliang Zhang^1^

*^1^ Wuhan National Laboratory for Optoelectronics, School of Optical and Electronic Information, Huazhong University of Science and Technology, Wuhan 430074, China*

*^2^Department of Electronic Engineering, The Chinese University of Hong Kong, Shatin, Hong Kong SAR, China*

^*^Corresponding author: [hailongzhou@hust.edu.cn](mailto:hailongzhou@hust.edu.cn); [jjdong@hust.edu.cn](mailto:jjdong@hust.edu.cn)

# S1 Partially coherent optical source and its application in computing

A partially coherent optical source is characterized by a relatively large linewidth and short coherence length. Unlike narrow-linewidth coherent lasers, which face challenges in stabilizing and maintaining high optical coherence, partially coherent optical sources, such as light-emitting diodes (LEDs) and amplified spontaneous emission (ASE) sources, are robust against phase noise and are simpler to generate and control. The electric field of a partially coherent source can be expressed as: , where *ω* is the center frequency, and *A*(*t*) and $\phi(t)$ are the fast-varying amplitude and phase noise, respectively. The power spectrum of *A*(*t*)e^i^*^ϕ^*(*^t^*) corresponds to the measured optical spectrum. When a partially coherent source is split into two channels, and the path difference exceeds the coherence length before the channels converge, the combined power can be expressed as (assuming the average amplitude of *A*(*t*) is one)^1^:

where *A*_1_ and *A*_2_ are the information loaded onto the two light channels, and *τ* (the latency between the two paths) is longer than the coherence time. This approximation holds true when the detection bandwidth (regarded as a cut-off response) is much lower than the optical spectrum bandwidth, that is^2^,

where *B*_e_ and *B*_o_≫*B*_e_ are detection bandwidth and optical linewidth respectively. *p*(*t*) represents the influence of source phase. In the equation, the terms marked in red are the detected noise and the rest two terms are constants. Consequently, as the linewidth of a partially coherent source *B_o_* increases, quality of the eye diagram (Fig. 5(b)) improves due to reduced noise proportion.

In a typical MZI mesh, different optical paths are designed to be nearly identical in length. As a result, partially coherent light maintains sufficient temporal coherence to interfere with itself within the mesh. In contrast, when partially coherent light enters different input ports, it first experiences significantly different optical path lengths, preventing mutual interference. To achieve broadband operation, all optical paths must maintain equal lengths. Based on the above features of partially coherent optical source, we can model the transmission matrix of MZI mesh under partial coherence illumination as **M**_pc_=|**M**_co_|^2^. **M**_pc_ is the intensity transmission matrix and **M**_co_ is the complex amplitude transmission matrix. |**M**_co_|^2^ denotes a matrix constituted by the modulus square of each element of **M**_co_.

# S2 Chip latency fitting

Fig.S1 (a-e) The simulated output pulse with different widths of input pulse for one O-E-O nonlinearity. (f) The measured reference pulse and output pulse from the second layer of NAFs. Dashed lines represent the fitted waveforms.

The input optical pulse is modeled as a Gaussian pulse:

$$P(t)=\text{e}^{-4t^{2}/\omega^{2}}$$

where *ω* represents the pulse width. The O-E-O nonlinearity is assumed to behave as a first-order system, with its impulse response given by:

$$h(t)=\left\{\begin{matrix} 0,t<0 \\ \text{e}^{-t/\tau},t\geq0 \end{matrix}\right.$$

where *τ* is the RC latency of NAF. Using this model, we fit the measured waveform in Fig. 2(e) and derive an RC latency of 1.67 ns. To investigate the influence of the input pulse width on the measured latency, we simulate the output pulse with varying input pulse widths for a single O-E-O response, as shown in Figs. S1(a–e). When the pulse width is much smaller than the RC latency (Fig. S1(a)), the output pulse closely resembles *h*(*t*), resulting in an ultra-low pulse latency dominated by the pulse width and pulse propagation time. As the input pulse becomes wider, the RC latency becomes more evident in the total pulse latency. Beyond the latency of the first nonlinear layer shown in Fig. 2(e), we also measure the output pulse from the second layer (Fig. S1(f)). The output pulse deviates slightly from the fitted waveform due to the nonlinear O-E-O process. Nevertheless, the measured latency of 2.85 ns aligns well with the simulation results.

# S3 Experiment results of two-classification

Fig. S2(a) illustrates the final layer of the PDONN, where we perform a 2-class classification task using the fashion MNIST dataset. The dataset consists of 100 images of T-shirts and trousers for training, and another 100 images for testing. The training process and confusion matrix are shown in Figs. S2(b-d).

The classification accuracy of the networks is as follows:

- **Real-valued optical neural network**: 100% for training and 95% for testing.
- **Positive optical neural network**: 97% for training and 94% for testing.
- **Partially coherent optical neural network**: 96% for training and 98% for testing.

The accuracies of the real-valued, positive, and partially coherent networks are similar on the training data, suggesting that the task is relatively simple. However, it is noteworthy that the partially coherent optical neural network outperforms the others on the test data, indicating its superior ability to generalize and lower susceptibility to overfitting compared to the coherent network.

Fig.S2 (a) The principle of 4-classification and 2-classification. The outputs of 2-classification are taken from the differential optical power of the last layer. (b-d) The results on the 2-classification of fashion images with the real-valued optical neural network, positive optical neural network, and partially coherent optical neural network.

# S4 Power Consumption Analysis

The power consumption of the system is derived from three main sources: the laser source, external electrical circuits, and the optical chip:

- **Laser Source**: The total input optical power is about **0.2 W**, and with a wall-plug efficiency of **3.1%** for the integrated ASE source^3^, the optical source contributes **6.45 W** to overall power consumption.
- **Phase Shifters**: The chip contains **136 phase shifters**, each consuming about **20 mW** for a π phase shift, resulting in a total of **2.72 W**. This power consumption could be eliminated with static-mode alternatives like phase-change material-based or MEMS phase shifters^4,5^.
- **Optical NAF**: With an MRM driving current of **0.1 mA** and a photodetector bias voltage of **3 V**, the optical NAF consumes **30.3 mW** (0.1 mA × 3 V × 101).
- **VOAs**: Loading input information via 64 VOAs consumes **0.96 W** (15 mW × 64).
- **External Electrical Circuits**: The power consumption of the external electrical circuits is calculated using the equation: *P*_DAC_×*N*_ps_+*P*_tr_×*N*_in_+*P*_re_×*N*_out_=**0.41 W**, where the parameters *P*_DAC_, *N*_ps_, *P*_tr_, *N*_in_, *P*_re_, and *N*_out_ are detailed in Table S1.

In total, the system's power consumption is **10.57 W**.

**Table S1. Parameters for power consumption computing**

| Parameter | Description | Value |
| --- | --- | --- |
| *P*_DAC_ | Power consumption of low-speed DAC | 5 µW (AD8802 from ANALOG DEVICES) |
| *N*_ps_ | Number of phase shifters | 136 |
| *P*_tr_ | Power consumption of the transmitter | 6.1 mW^6^ |
| *N*_in_ | Input data size | 64 |
| *P*_re_ | Power consumption of the receiver | 5.4 mW^7,8^ |
| *N*_out_ | Output data size | 3 |

# References

1 Wu, B. *et al.* Chip-encoded high-security classical optical key distribution. *Nanophotonics* **13**, 3717-3725 (2024). <https://doi.org/doi:10.1515/nanoph-2024-0188>

2 Dong, B. W. *et al.* Partial coherence enhances parallelized photonic computing. *Nature* **632**, 55-62 (2024). <https://doi.org/10.1038/s41586-024-07590-y>

3 Mehta, K. *et al.* High-Power Heterogeneously Integrated III-V/Silicon Superluminescent Diode. *IEEE Photonics Technology Letters* **35**, 365-368 (2023). <https://doi.org/10.1109/LPT.2023.3245950>

4 Wei, M. *et al.* Electrically programmable phase-change photonic memory for optical neural networks with nanoseconds in situ training capability. *Advanced Photonics* **5** (2023). <https://doi.org/10.1117/1.Ap.5.4.046004>

5 Errando-Herranz, C. *et al.* MEMS for Photonic Integrated Circuits. *Ieee Journal of Selected Topics in Quantum Electronics* **26**, 1-16 (2020). <https://doi.org/10.1109/Jstqe.2019.2943384>

6 Kim, J. *et al.* in *2017 IEEE Radio Frequency Integrated Circuits Symposium (RFIC).* 248-251 (Proceedings of the IEEE, 2017).

7 Abd-elrahman, D., Atef, M., Abbas, M. & Abdelgawad, M. in *2015 IEEE International Conference on Electronics, Circuits, and Systems (ICECS).* 244-247 (Proceedings of the IEEE, 2016).

8 Oh, D. R. *et al.* in *2020 IEEE Symposium on VLSI Circuits.* 1-2 (Proceedings of the IEEE, 2020).
